# Irradiation pretreatment enhances the therapeutic efficacy of platelet-membrane-camouflaged antitumor nanoparticles

**DOI:** 10.1186/s12951-020-00660-z

**Published:** 2020-07-20

**Authors:** Yin Chen, Xue Shen, Songling Han, Tao Wang, Jianqi Zhao, Yongwu He, Shilei Chen, Shengqi Deng, Cheng Wang, Junping Wang

**Affiliations:** 1 State Key Laboratory of Trauma, Burns and Combined Injury, Institute of Combined Injury of PLA, Chongqing Engineering Research Center for Nanomedicine, College of Preventive Medicine, Third Military Medical University, Chongqing, 400038 China; 2 Sichuan Industrial Institute of Antibiotics, Chengdu University, Chengdu, 610106 China; 3 College of Materials Science and Engineering, Hebei University of Engineering, Handan, 056038 China

## Abstract

**Background:**

Cell membrane-based nanocarriers are promising candidates for delivering antitumor agents. The employment of a simple and feasible method to improve the tumor-targeting abilities of these systems is appealing for further application. Herein, we prepared a platelet membrane (PM)-camouflaged antitumor nanoparticle. The effects of irradiation pretreatment on tumor targeting of the nanomaterial and on its antitumor action were evaluated.

**Results:**

The biomimetic nanomaterial constructed by indocyanine green, poly(d,l-lactide-co-glycolide), and PM is termed PINPs@PM. A 4-Gy X-ray irradiation increased the proportions of G2/M phase and Caveolin-1 content in 4T1 breast cancer cells, contributing to an endocytic enhancement of PINPs@PM. PINPs@PM produced hyperthermia and reactive oxygen species upon excitation by near-infrared irradiation, which were detrimental to the cytoplasmic lysosome and resulted in cell death. Irradiation pretreatment thus strengthened the antitumor activity of PINPs@PM in vitro. Mice experiments revealed that irradiation enhanced the tumor targeting capability of PINPs@PM in vivo. When the same dose of PINPs@PM was intravenously administered, irradiated mice had a better outcome than did mice without X-ray pretreatment.

**Conclusion:**

The study demonstrates an effective strategy combining irradiation pretreatment and PM camouflage to deliver antitumor nanoparticles, which may be instrumental for targeted tumor therapy.

## Background

Cancer is a global threat to human health [1]. Surgery, radiotherapy, and chemotherapy are conventional methods to treat cancers, but they all have inherent limitations in clinical applications, such as invasiveness, drug resistance, and severe side effects [2]. Phototherapy, including photothermal therapy (PTT) and photodynamic therapy (PDT), is a noninvasive and effective antineoplastic strategy and is considered a promising alternative to classical oncotherapy [3]. PTT and PDT eliminate cancer cells based on the fact that after excitation with light of a specific wavelength, photothermal agents and photosensitizers generate hyperthermia and reactive oxygen species (ROS), respectively, which are detrimental to cancer cells [4, 5].

Indocyanine green (ICG), one of the near-infrared dyes approved by the US Food and Drug Administration for clinical imaging and diagnosis, is a photothermal agent as well as a photosensitizer and thus attracts considerable attention. Because ICG lacks a tumor-targeting ability and tends to be rapidly cleared in vivo, many nanocarriers have been developed to deliver ICG [6]. Nevertheless, provided that the carrier is not endowed with antiphagocytic ability against the mononuclear phagocyte system in vivo, the bioavailability of ICG is still limited [7]. Cell membrane-based nanoparticles (CMBNPs) represent promising materials to overcome this shortcoming [8], as the functional molecules on the membrane, such as CD47 [9], CD45, and glycans, can send a “don’t eat me” signal to the immune system [10]. The biomimetic strategy is plausibly beneficial for ICG delivery.

Intensifying the tumor-targeting ability of nanocarriers is also instrumental for ICG delivery. Passive and active targeting strategies are employed by therapeutic nanoparticles (TNPs) to reach the tumor site. Compared with the passive targeting phenomenon, which is mostly based on the enhanced permeability and retention effect and is limited by tumor types [11, 12], the active targeting method, such as modifying TNPs with peptides and antibodies [13, 14], is more efficient to promote drug accumulation in tumors. Notably, due to the presence of functional molecules with high affinity to cancer cells on certain cell membranes, P-selectin on the platelet membrane (PM) [4], for example, some CMBNPs possess an active tumor targeting capability. Further decoration of the cell membrane with tumor necrosis factor-related apoptosis-inducing ligand can strengthen the targeting ability [15], but the process is somewhat complex. The employment of simple and feasible methods that can help to target these CMBNPs to the tumor site is appealing for future clinical use.

**Fig. 1:**
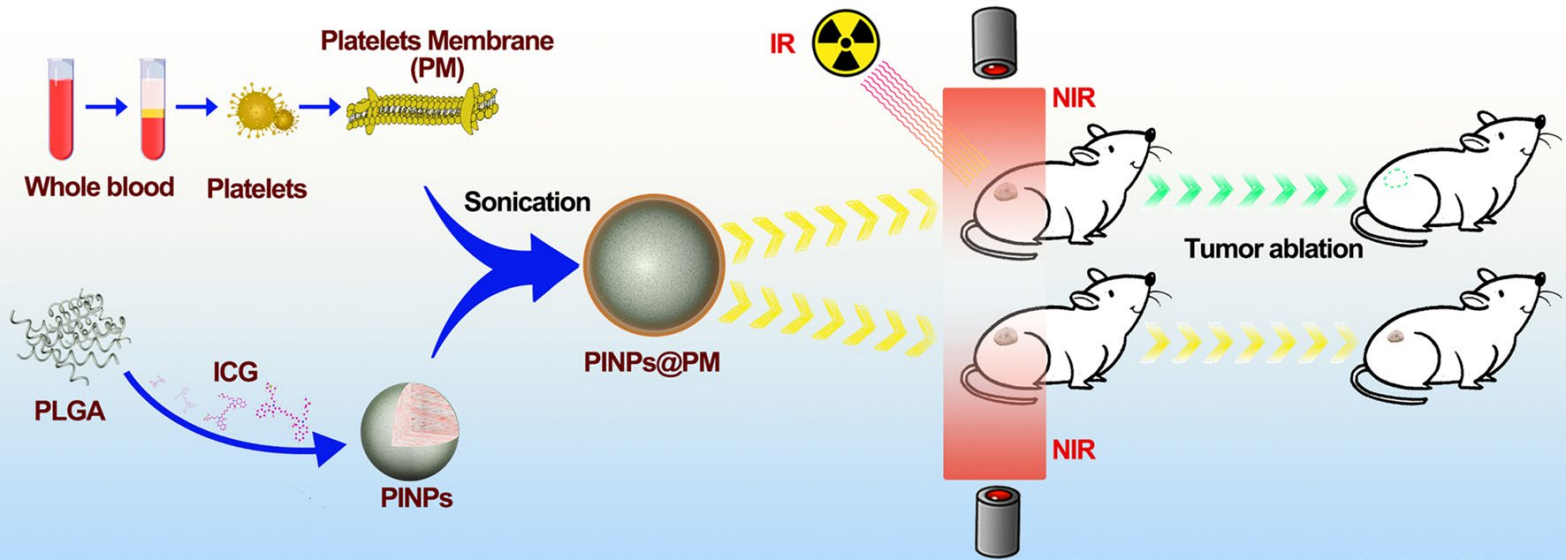
Diagrammatic drawing depicting the preparation and evaluation of PINPs@PM

In this study, we employed a PM-camouflaged poly(d,l-lactide-co-glycolide) (PLGA) nanocarrier to deliver ICG, obtaining a composite termed PINPs@PM (Fig. 1). The effect of X-ray irradiation on the antitumor activity of PINPs@PM in the presence of near-infrared irradiation (NIR) in vitro and in vivo was evaluated. The combined strategy using irradiation pretreatment and cell membrane camouflage is efficient for delivering drugs to tumors and thus has wide prospects in tumor diagnosis and therapy.

## Results and discussion

### Preparation and characterization of PINPs@PM

**Fig. 2:**
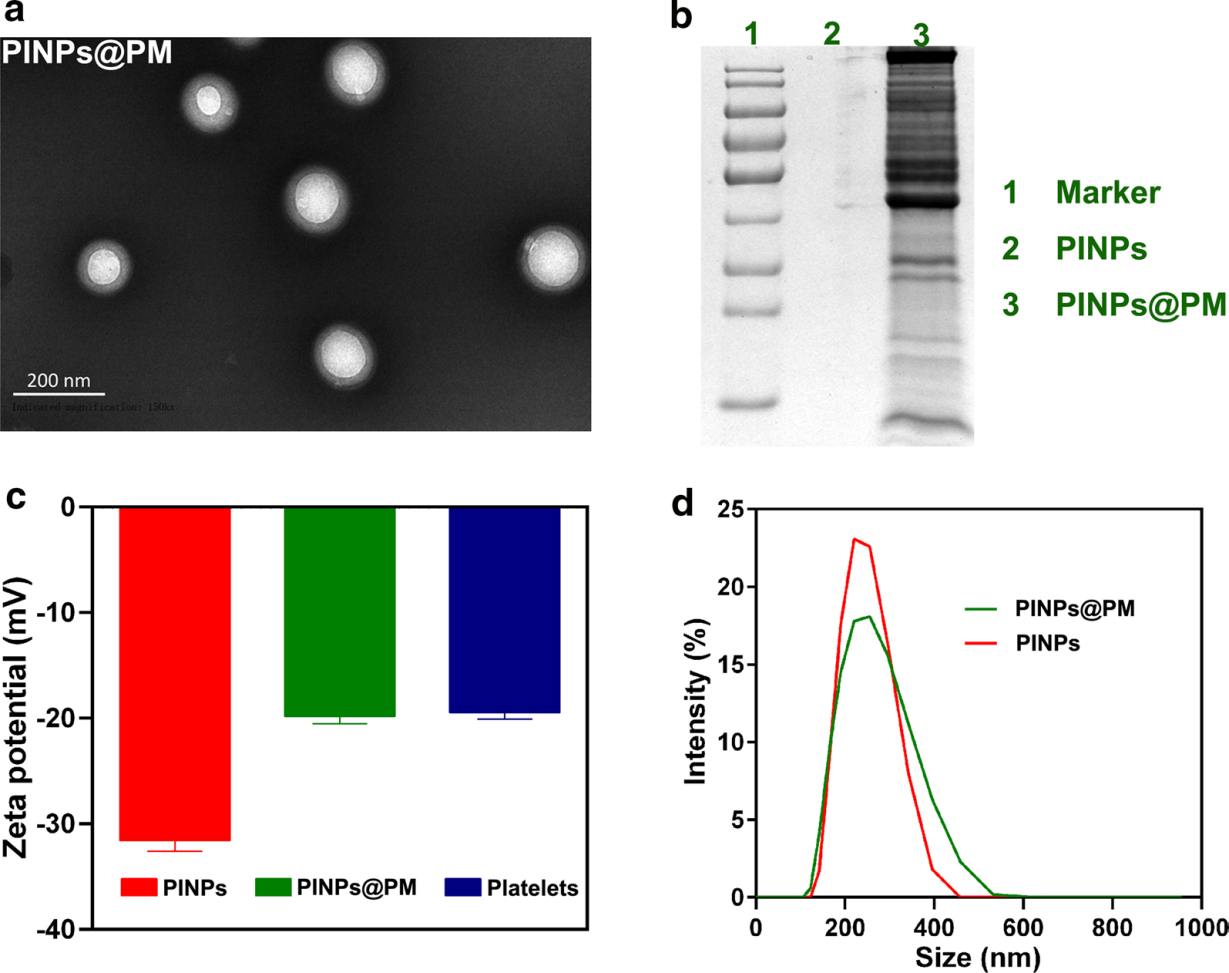
Characterization of PINPs@PM. **a** TEM image of PINPs@PM. The scale is 200 nm. **b** SDS-PAGE analysis. The PM protein (20 μg) was resolved by 10% SDS-PAGE. **c** Surface charge determination. The results are shown as the mean ± SD. **d** DLS detection of the hydrodynamic diameters

The ICG-loaded PLGA nanoparticles fabricated using a double emulsion method were termed PINPs (Fig. 1). The encapsulation efficiency of ICG was 60.6%, calculated by ultraviolet spectrophotometry as we recently described [16]. PINPs were spherical with a good dispersion ability (Additional file 1). After decoration with a PM, the nanoparticles became PINPs@PM which exhibited white halos by transmission electron microscopy (TEM, Fig. 2a). Sodium dodecyl sulfate–polyacrylamide gel electrophoresis (SDS-PAGE) confirmed the successful coating of PM (Fig. 2b) [17]. BCA assay indicated that the average coating efficiency of PM on PINPs was 57.3%. The zeta potential determination revealed that the PM camouflage increased the surface charge of the PINPs (averagely − 31 mV) to approximately − 20 mV, which was similar to the charge of free platelets (Fig. 2c). The hydrodynamic diameter of the PINPs@PM (Size = 255 nm, PDI = 0.143) measured by dynamic laser scattering (DLS) was slightly larger than that of the PINPs (Fig. 2d, Size = 219 nm, PDI = 0.118), which was consistent with the influence of red blood cell membrane cloaking on PLGA nanoparticles encapsulating perfluorocarbons [18].

### In vitro and in vivo toxicological evaluation

**Fig. 3:**
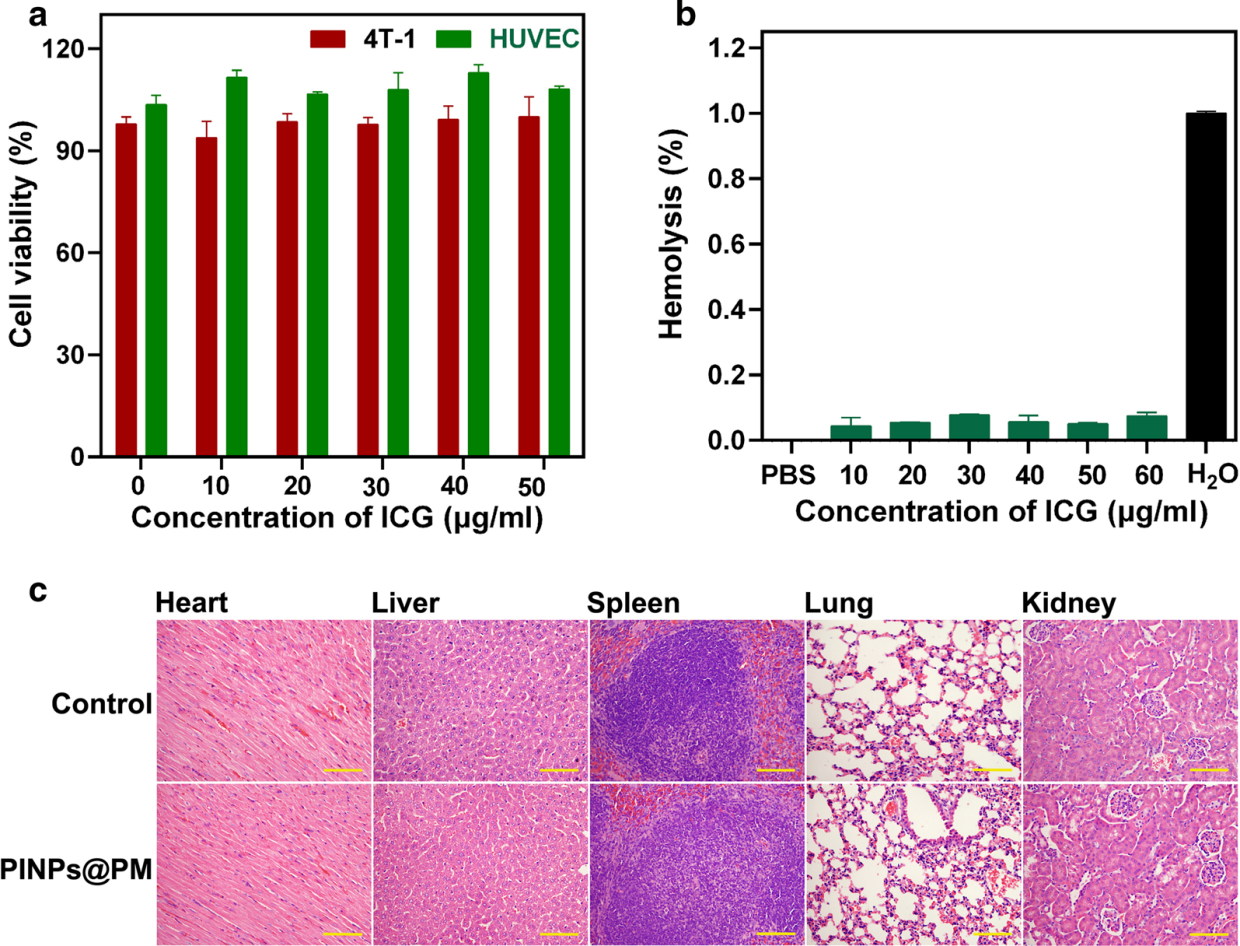
Toxicity evaluation of PINPs@PM. **a** The survival of HUVECs and 4T1 cells exposed to different concentrations of PINPs@PM for 24 h. The results are shown as the mean ± SD. **b** Haemolysis of different concentrations of PINPs@PM. **c** HE staining of the organs of mice treated with NaCl and PINPs@PM. The scale is 100 μm

To assess the material toxicity, we incubated PINPs@PM with human umbilical vein endothelial cell (HUVEC) and mouse 4T1 cells for 24 h. PINPs@PM had less of an effect on the cell survival at concentrations up to 50 μg/ml (based on the concentration of ICG, Fig. 3a). The marginal haemolytic property of PINPs@PM further confirmed its biosafety in vitro (Fig. 3b). Animal experiments showed that intravenous injection of 60 μg of PINPs@PM (300 μg/ml, based on the dose of ICG) was nonlethal to BALB/c mice. The vital organs including the heart, liver, spleen, lung, and kidney were obtained after 3 weeks. In line with the nontoxicity of CMBNPs [19], there were no pathological changes in the tissue revealed by haematoxylin and eosin (HE) staining (Fig. 3c), indicative of the biocompatibility of PINPs@PM in vivo.

### Effect of irradiation on the endocytosis of PINPs@PM by cancer cells

**Fig. 4:**
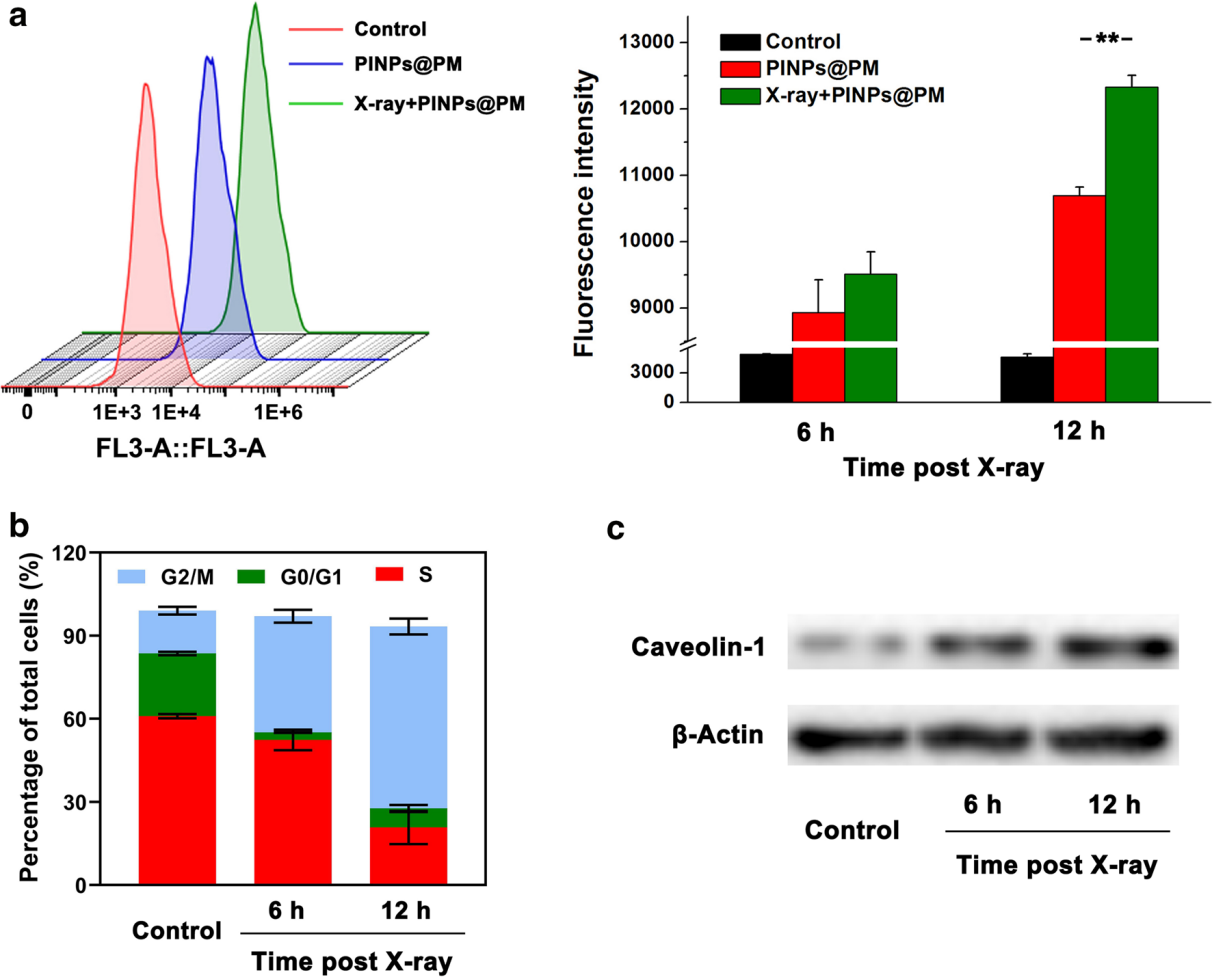
Cellular uptake of PINPs@PM post irradiation. **a** Flow cytometry of 4T1 cells treated with PINPs@PM for 12 h in the presence and absence of irradiation pretreatment. The fluorescence intensity is presented as the mean ± SD. **, *P* < 0.01. **b** Cell cycle distribution of 4T1 cells treated with 4-Gy X-ray irradiation. The proportions of each phase are presented as the mean ± SD. **c** Immunoblotting revealed the alterations of Caveolin-1 in 4T1 cells after irradiation. β-actin was employed as the reference

Platelets have pathophysiological affnity with tumors [20]. The interaction between P-selectin and CD44 partially contributes to the binding of PM-camouflaged nanomaterials to the surface of breast cancer cells [4, 21]. After aggregation on the cell membrane, our data showed that PINPs@PM was endocytosed by 4T1 cells in a time-dependent manner. Notably, pretreatment of the cells with a 4-Gy X-ray irradiation (1.0 Gy/min) enhanced the endocytosis without inducing severe membrane damage (Additional file 2). Flow cytometry revealed that the fluorescence intensity of 4T1 cells phagocytosing 30 μg/ml PINPs@PM after 12 h averaged 12,332, which was significantly higher than that of cells without irradiation (average of 10,696, Fig. 4a). We measured the cell cycle of 4T1 cells 12 h post irradiation and found that the proportions of G2/M phase and G0/G1 phase cells were increased from 15.43% to 65.63% and decreased from 22.6 to 6.85%, respectively (Fig. 4b). Additionally, Caveolin-1, a membrane protein to constitute caveolae [22], was increased post irradiation (Fig. 4c). Because cells in the G2/M and G0/G1 phases are the most efficient and inefficient to uptake nanoparticles [23], and because over-expression of Caveolin-1 benefits material endocytosis by cancer cells [24], the alterations in cell cycle and Caveolin-1 content we considered contributed to the enhancement effect of X-ray irradiation on the uptake of PINPs@PM by 4T1 cells.

### Antitumor evaluation in vitro

**Fig. 5:**
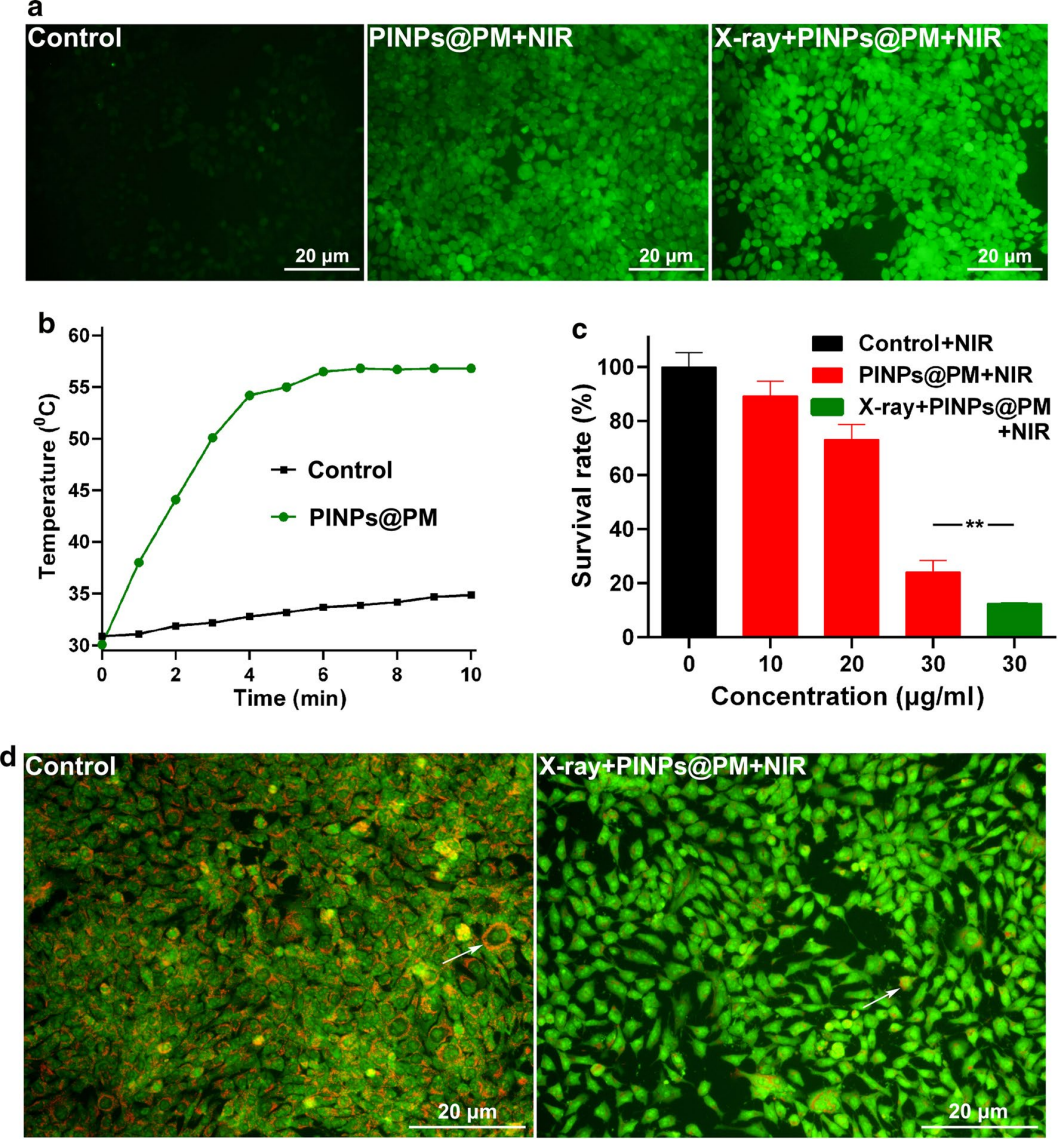
Irradiation pretreatment strengthens the antitumor activity of PINPs@PM in vitro. **a** ROS staining of 4T1 cells phagocytosing PINPs@PM in the presence and absence of X-ray irradiation. The scale is 20 μm. **b** The temperature of PINPs@PM (30 μg/ml) upon excitation by NIR for different times. **c** The survival of 4T1 cells treated with PINPs@PM in the presence and absence of irradiation pretreatment. **, *P* < 0.01. **d** AO staining showing the damage of lysosome in 4T1 cells. White arrows indicate the representative cytoplasmic lysosome. The scale is 20 μm

ICG is a photosensitizer and a photothermal agent. After the endocytosis by 4T1 cells, PINPs@PM produced intracellular ROS that could be stained by 2′,7′-dichlorodihydrofluorescein diacetate (DCFH-DA) probes upon excitation by NIR at 808 nm (Fig. 5a). The green fluorescence of X-ray-treated cells was visually stronger than that of cells without irradiation, which also indirectly indicated that irradiation pretreatment could strengthen the endocytosis of PINPs@PM by cancer cells. ROS are closely related to the growth and death of cancer cells [25]. On the one hand, ROS can induce the DNA mutation and genomic instability, accelerating the proliferation, immune tolerance, and metastasis of cancer cells [26, 27]. In addition, high ROS levels enhance the cellular oxidative stress, which is detrimental to DNA, proteins, and lipids and thus causes cell death [28]. Ample studies have exploited the modulation of oxidative stress by delivering photosensitizers to the tumor site as the therapeutic strategy [29–31].

Otherwise, the temperature of PINPs@PM increased with increasing exposure time to NIR (Fig. 5b). After 10 min of NIR (808 nm, 1 W/cm^2^), the temperature of 30 μg/ml PINPs@PM reached 56.8 °C, which is sufficient to damage proteins and inactivate cancer cells [32]. Accordingly, with the actions of excess ROS and hyperthermia, the 4T1 cells were eliminated in a dose-dependent manner by PINPs@PM (Fig. 5c). Similar to the endocytic tendency shown in Fig. 4a, X-ray irradiation pretreatment significantly intensified the antitumor activity of PINPs@PM in vitro.

Lysosomes are considered therapeutic targets to treat many diseases including rheumatoid arthritis, Alzheimer’s disease, and cancers [33, 34]. Lysosome is the intracellular target of some nanomaterials and its permeabilization is involved in material-mediated cell death [35]. Inhibiting lysosome function enhances the antitumor activity of chemotherapeutic drugs and increases the sensitivity of tumors to radiotherapy [36, 37]. We employed the acridine orange (AO) dye to stain 4T1 cells. Normally, the intact acidic lysosome exhibits red fluorescence, while the cytosol and nuclei exhibit green fluorescence upon excitation at 488 nm. Fluorescence microscopy showed that the red fluorescence in cells treated with X-ray/PINPs@PM/NIR was dramatically decreased compared with that in PBS-treated cells (Fig. 5d), suggesting that the lysosome is severely injured by the combined treatment. Due to the photochemical internalization effect of ROS on the membrane of lysosomes and the photothermal effect of ICG, the ROS and hyperthermia generated by NIR-treated PINPs@PM may be responsible for the lysosome disruption [38], which partially contributes to cell death.

### Tumor targeting and therapeutic assessment in vivo

**Fig. 6:**
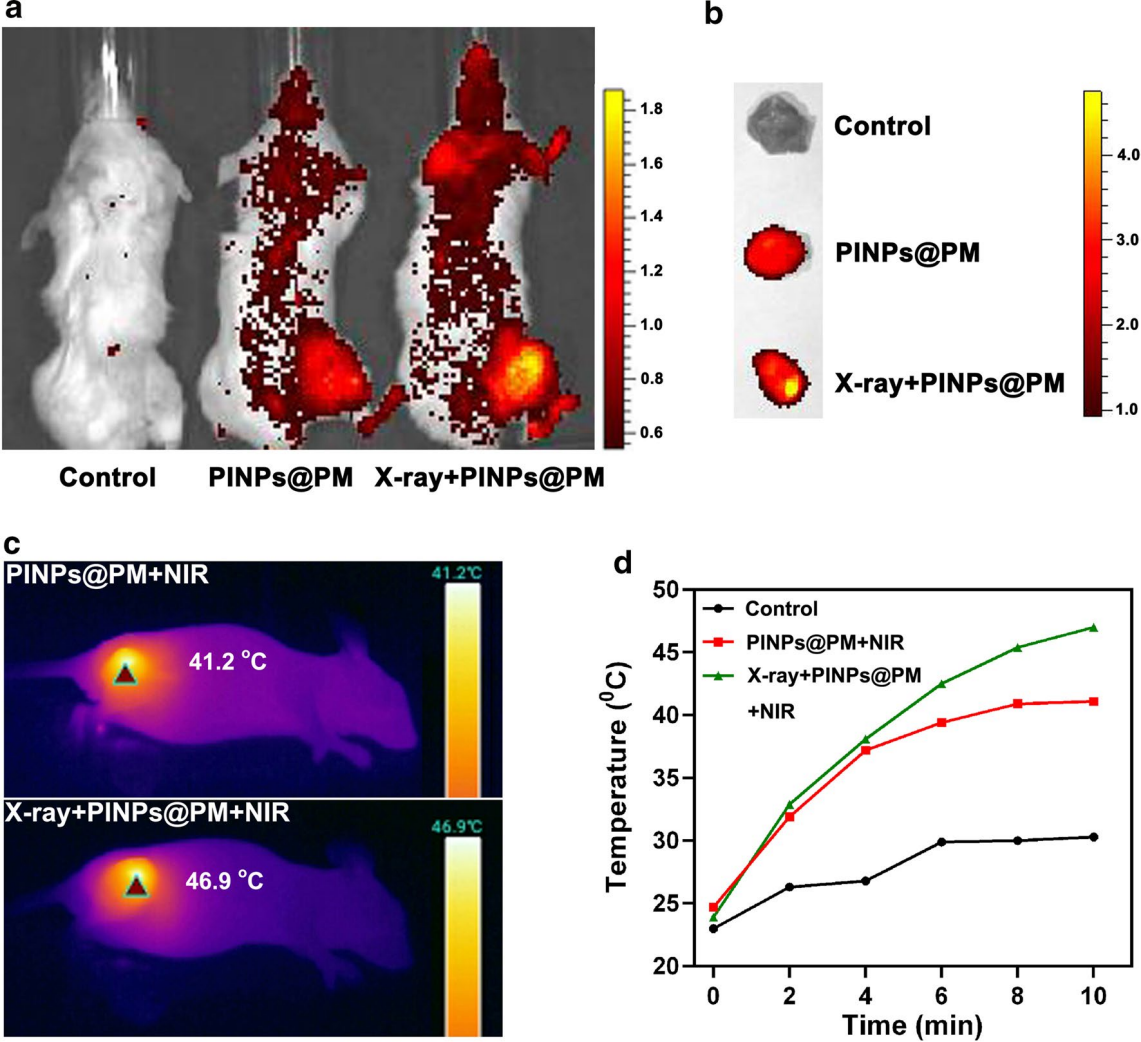
Irradiation pretreatment enhances the accumulation of PINPs@PM in the tumor site. **a** In vivo imaging of tumor-bearing mice at 24 h post injection of PINPs@PM. **b** In vitro imaging of PINPs@PM in tumors. **c** Infrared thermal images of tumor-bearing mice treated with PINPs@PM in the presence and absence of X-ray irradiation. **d** The maximal tumor temperature of PINPs@PM-treated mice after NIR treatment for different times

We obtained the fluorescent images of 4T1 tumor-bearing mice intravenously administered with 60 μg of PINPs@PM 12 h (Additional file 3) and 24 h (Fig. 6a) post irradiation. Because irradiation not only enhances the material endocytosis by cancer cells but also enables the alteration of the tumor microenvironment to accumulate nanoparticles [39], PINPs@PM accumulated more at the tumor site with the assistance of X-ray than did the group without irradiation pretreatment, which was also evidenced by the in vitro tissue imaging (Fig. 6b). The infrared thermal imaging supported that the maximal tumor temperature of PINPs@PM-treated mice reached 41.2 °C after 10 min of NIR treatment (Fig. 6c), which was much higher than that of NaCl-treated mice (30.3 °C, Additional file 4) but lower than that of X-ray-pretreated mice (46.9 °C). In contrast to the PINPs@PM/NIR group whose temperature tended to balance after 8 min, the X-ray/PINPs@PM/NIR group exerted an overt time-dependent temperature increase (Fig. 6d).

**Fig. 7:**
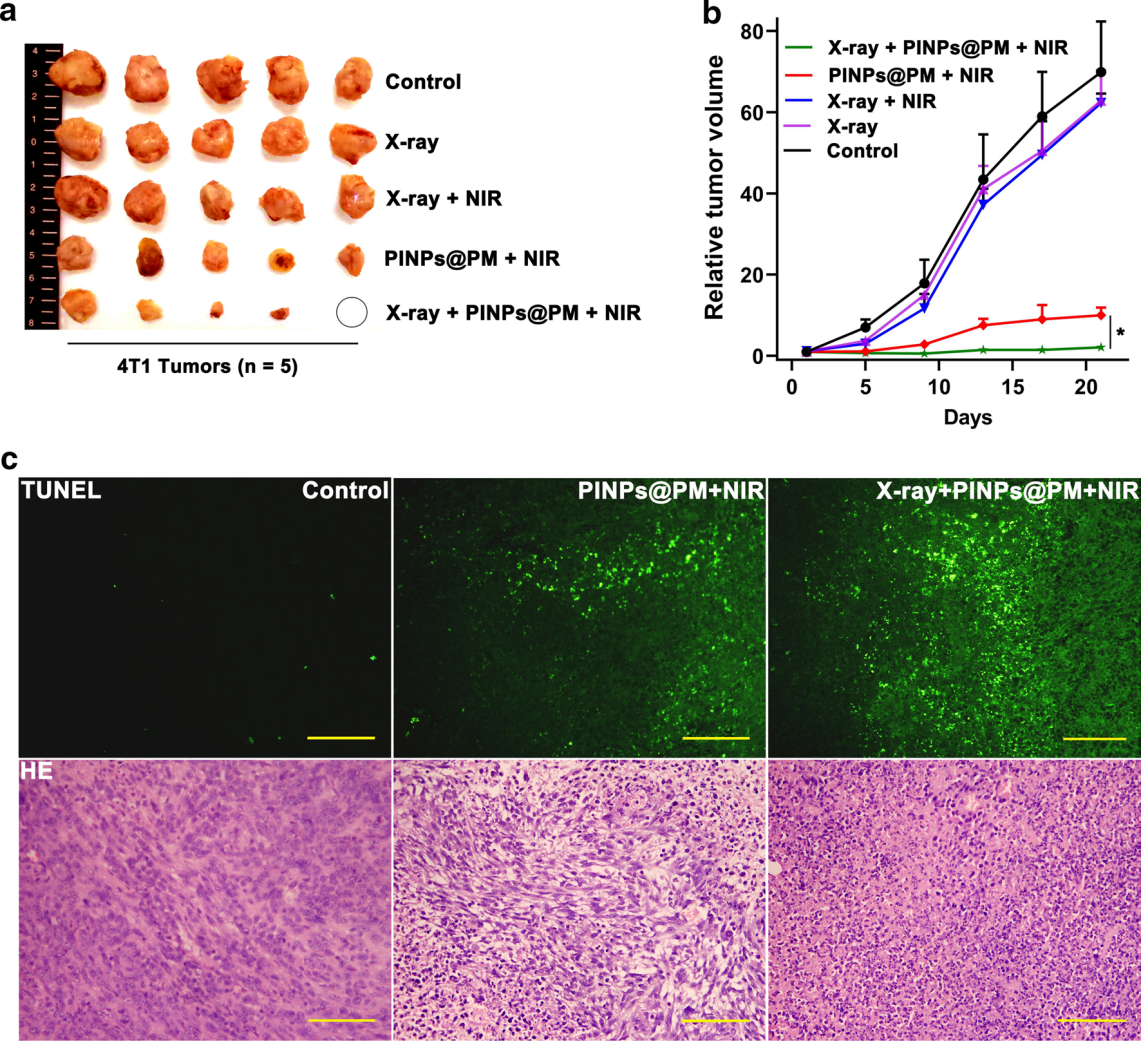
Irradiation pretreatment improves the antitumor ability of PINPs@PM in vivo. **a** Photograph of mice tumors (n = 5). The circle indicates that the tumor is visually eliminated. **b** Dynamic alterations of tumor volumes. *, *P* < 0.05. **c** TUNEL and HE staining showing the pathological changes of tumor tissues. The scale is 100 μm

After NIR treatment, mice were monitored for 21 days and sacrificed, and the tumors were obtained by surgery (Fig. 7a). Consistent with that PM cloaking improves the therapeutic efficacy of mesoporous silica-coated bismuth nanorod as we recently described [4], PINPs@PM was superior to PINPs at decreasing the tumor volumes (Additional file 5). Due to the enhancement to tumor targeting, irradiation pretreatment further improved the curative effect of PINPs@PM, as revealed by the significantly lowered tumor sizes in both volume (Fig. 7b) and mass (Additional file 6). Terminal deoxynucleotidyl transferase-mediated deoxyuridinetriphosphate nick end labeling (TUNEL) assay showed that the dead cells in X-ray/PINPs@PM/NIR-treated tumors are visibly more than those in tumors without irradiation pretreatment (Fig. 7c). HE staining indicated that the structure of tumor tissue was severely destroyed after X-ray/PINPs@PM/NIR treatment.

## Conclusions

ICG-based phototherapy is an effective strategy to treat tumors, whereas the circulation time of free ICG is short. In this study, we prepared a PM-camouflaged PLGA nanoparticle to deliver ICG, considering that PM camouflage not only lowers the immune clearance of nanocarriers but strengthens their tumor targeting capabilities. For further targeting enhancement, we optimized the therapeutic regimen by adding an irradiation pretreatment. The X-ray irradiation increased endocytosis of the nanomaterial by cancer cells and enhanced its targeting efficiency, thus intensifying its antitumor action in vitro and in vivo. The study demonstrating a combined use of irradiation pretreatment and PM camouflage to improve the targeting ability of antitumor nanoparticles is useful for further tumor therapy.

## Methods

### Materials

PLGA (50:50, inherent viscosity 0.20 dl/g, MW = 15,000) was obtained from the Shandong Institute of Medical Instruments (Jinan, Shandong Province, CHN). Poly(vinyl alcohol) (PVA, MW = 30,000–70,000, 78–90% hydrolysed) and ICG were purchased from Sigma-Aldrich (St Louis, MO, US). RPMI 1640 cell culture medium and foetal bovine serum (FBS) were obtained from Gibco (Thermo Fisher Scientific, Shanghai, CHN). The SDS-PAGE, DCFH-DA, and CCK-8 kits were obtained from Beyotime (Shanghai, CHN). The formaldehyde solution (4%), HE staining solution, and AO were purchased from Solarbio (Beijing, CHN). Deionized water was obtained from a Milli-Q water purification system.

### Preparation of PINPs and PINPs@PM

The ICG-loaded PLGA nanoparticles (PINPs) were prepared using a reported water-in-oil-in-water double emulsion method with modifications [16, 40]. Briefly, PLGA (120 mg) was dissolved in 2.5 ml of methylene chloride. ICG solution (7.5 mg/ml, 200 μl) was added, and the mixture was emulsified by sonication (25% amplitude, 2 min) using a Digital Sonifier S-250D (Branson Ultrasonic, CT, US) in an ice bath. The primary emulsion (first emulsion) was poured into 10 ml of PVA solution (3%, w/v) and sonicated for another 3 min, obtaining a double emulsion (second emulsion). The obtained product was stirred to evaporate the organic solvent and was then centrifuged at 1200 rpm for 12 min to remove the non-encapsulated ICG.

PM camouflage was performed as we recently described [4]. Mouse platelets were collected by gradient centrifugation, frozen at − 80 °C and thawed at room temperature. After three freeze–thaw cycles, the membrane was obtained by centrifugation at 8000 rpm for 10 min, washed with PBS containing protease inhibitor and sonicated for 5 min. PINPs (300 μg, based on the content of ICG) dissolved in PBS (1 ml) were added to equal volumes of PM. The mixture was then sonicated on ice for 2 min and maintained at 4 °C overnight. Large fragments of PM were removed using a filter membrane with a pore size of 400 nm. The PINPs@PM was collected by high-speed centrifugation at 10,000 rpm for 15 min. A BCA assay was employed to determine the efficiency of membrane coating. Animals were cared for and treated in accordance with the National Institutes of Health (NIH) guidelines for the care and use of laboratory animals (NIH Publication No. 85e23 Rev. 1985) as approved by the Animal Experimental Ethics Committee of TMMU.

### Characterization analysis

The morphologies of the PINPs and PINPs@PM were obtained by TEM (Tecnai G2 F20 U-TWIN, FEI, Hillsboro, OR, US). To confirm the PM camouflage, PINPs@PM was denatured and resolved via 10% SDS-PAGE. The protein bands were visualized by Coomassie blue staining. The DLS and zeta potential experiments were determined by a Nano-ZS (Malvern, Worcestershire, UK) at room temperature. The temperature alteration of PINPs@PM exposed to NIR at 808 nm for different times (1.0 W/cm^2^, Laserwave, Beijing, CHN) was recorded with a thermoelectric thermometer (HH806W, Omega, US).

### Toxicological evaluation

HUVECs and 4T1 cells obtained from the Chinese Academy of Sciences (Shanghai, CHN) were cultured in RPMI 1640 medium containing 10% FBS. Ten thousand cells cultured to the logarithmic phase were incubated with different concentrations of PINPs@PM (0, 10, 20, 30, 40, and 50 μg/ml, based on the dose of ICG) for 24 h. The cell viability was determined by CCK-8 assay. Mouse erythrocytes diluted in saline solution (2%, v/v, 300 μl) were incubated with 1.2 ml of PINPs@PM at 37 °C for 2 h. The absorbance of the supernatant was determined at 405 nm. These experiments were conducted in triplicate and repeated twice. The toxicity of PINPs@PM in vivo was evaluated by a mouse experiment. Animals were cared for and treated as demonstrated in the preparation of PINPs@PM. PINPs@PM (60 μg, based on the content of ICG) was intravenously injected. The mice were sacrificed after 7 days, and their hearts, livers, spleens, lungs, and kidneys were obtained by surgery. The pathological changes were observed with an Olympus DX51 optical microscope (Tokyo, JPN) after HE staining.

### Flow cytometry-based endocytic assay

4T1 cells were seeded in a 6-well plate at a density of 2 × 10^5^ CFU per well and cultured overnight in RPMI 1640 medium containing 10% FBS. PINPs@PM (30 μg/ml, based on the content of ICG) was co-incubated with 4T1 cells for 6 and 12 h in the presence and absence of a 4-Gy X-ray irradiation pretreatment with an RS2000 X-ray irradiator (1.0 Gy/min, Rad Source, Suwanee, GA). The counts of fluorescent cells uptaking ICG-loaded PINPs@PM excited at 780 nm and the fluorescence intensity were determined with a BD flow cytometry system (Franklin Lakes, NJ, US), in which 15,000 events per sample were obtained. The experiment was repeated three times, and the data were processed using FlowJo software (version 7.6.1).

### Cell cycle analysis

4T1 cells (2 × 10^5^ CFU) were cultured overnight in a 6-well plate and irradiated with a 4-Gy X-ray. After further incubation in RPMI 1640 medium containing 10% FBS for 6 and 12 h, cells were harvested by trypsinization and fixed with 70% ethanol at 4 °C for 24 h. The cells were then stained with PI for 30 min after resuspension in RNase A buffer. The cell cycle was determined with a BD C6 flow cytometer. The data were processed using FlowJo software (version 7.6.1). This experiment was repeated three times on different days.

### Western blot

4T1 cells irradiated with a 4-Gy X-ray and incubated for 6 and 12 h were collected and processed with RIPA lysis and extraction buffer (89,900, Thermo Fisher Scientific). The cell extracts were denatured and resolved via 10% SDS-PAGE. A primary anti-Caveolin-1 rabbit monoclonal antibody (Abcam, ab32577, 1:200, Shanghai, CHN) and a goat anti-rabbit secondary antibody (Abcam, ab205718, 1:1000) were employed to detect Caveolin-1. β-actin determined by a mouse monoclonal antibody (AA128, Beyotime, 1:1000) was used as a reference. This assay was repeated three times on different days.

### Antitumor cell experiment

4T1 cells seeded in a 96-well plate at a density of 1 × 10^4^ CFU per well were cultured overnight and incubated with 30 μg/ml of PINPs@PM (based on the dose of ICG) in the presence and absence of a 4-Gy X-ray irradiation pretreatment. NIR (808 nm, 1.0 W/cm^2^, 10 min) was performed after 12 h of co-incubation. Cells were then washed with sterile PBS, stained with 100 μl of DCFH-DA (10 μM) and AO dyes (5 μg/ml) according to the manufacturer’s instructions and observed with an Olympus IX70 inverted fluorescence microscope. Otherwise, the cells were further incubated for 16 h followed by NIR treatment and the survival was detected by a CCK-8 assay. The experiment was conducted in duplicate and repeated three times.

### Tumor targeting evaluation

Female 8-week-old BALB/c mice were subcutaneously injected with 2 × 10^9^ CFU 4T1 cells in the right hind leg. Animals were cared for and treated as demonstrated in the preparation of PINPs@PM. A 4-Gy X-ray local irradiation was performed when the tumor reached approximately 75 mm^3^. PINPs@PM (60 μg, based on the content of ICG) was intravenously administered 6 h later. The accumulation of PINPs@PM in the tumor-bearing mice was observed with an IVIS spectrum in vivo imaging system (Perkin Elmer, Shanghai, CHN) at 12 and 24 h post-treatment. A Fortric 226 s thermal imager (Shanghai, CHN) was used to obtain the infrared thermal images and record the temperature variations of the tumors along with the increasing NIR exposure times.

### Antitumor assay in vivo

The tumor-bearing mice (n = 5) were prepared and cared for as described in the tumor targeting evaluation. PINPs@PM (60 μg, based on the content of ICG) was given by tail vein injection in the presence and absence of a 4-Gy X-ray local irradiation. NIR treatment was continued for 10 min after 24 h. The tumor volumes were monitored for 3 weeks. The tumor tissues were obtained by surgery for weigh and TUNEL and HE staining.

### Statistical analysis

The significant differences (P) were calculated by SPSS 17.0 using the Student’s two-tailed t test and LSD multiple-comparison test. A P value lower than 0.05 was defined as statistically significant.

## Supplementary information

**Additional file 1.** TEM image of PINPs.

**Additional file 2.** 1-*N*-phenylnaph-thylamine (NPN)-uptake assay.

**Additional file 3.** In vivo imaging of tumor-bearing mice at 12 h post injection of PINPs@PM.

**Additional file 4.** Thermal images of NaCl-treated tumor-bearing mice.

**Additional file 5.** PM camouflage improves the antitumor ability of PINPs in vivo.

**Additional file 6.** Histogram showing the tumor weight at day 21.

## Data Availability

All data generated or analyzed during this study are included in this published article.
