# Supplementary material for: Irradiation pretreatment enhances the therapeutic efficacy of platelet-membrane-camouflaged antitumor nanoparticles
Source: J Nanobiotechnology. 2020 Jul 20;18:101. doi: 10.1186/s12951-020-00660-z (PMC7372815; doi:10.1186/s12951-020-00660-z)
Supplement: Supplementary file 1 — Additional file 1. TEM image of PINPs. [file 12951_2020_660_MOESM1_ESM.docx]

Additional File 1

Irradiation pretreatment enhances the therapeutic efficacy of platelet-membrane-

camouflaged antitumor nanoparticles

Yin Chen^1#^, Xue Shen^2#^, Songling Han^1^, Tao Wang^1^, Jianqi Zhao^1^ , Yongwu He^1, 3^, Shilei Chen^1^, Shengqi Deng^2^, Cheng Wang^1*^ and Junping Wang^1*^

^1^ State Key Laboratory of Trauma, Burns and Combined Injury, Institute of Combined Injury

of PLA, Chongqing Engineering Research Center for Nanomedicine, College of Preventive

Medicine, Third Military Medical University, Chongqing, 400038, China

^2^ Sichuan Industrial Institute of Antibiotics, Chengdu University, Chengdu, 610106, China

^3^ College of Materials Science and Engineering, Hebei University of Engineering, Handan,

056038, China

^#^ These authors contributed equally to this work.

^*^ Corresponding authors.

Junping Wang, [wangjunping@tmmu.edu.cn](mailto:wangjunping@tmmu.edu.cn); Cheng Wang, wangctmmu@126.com.


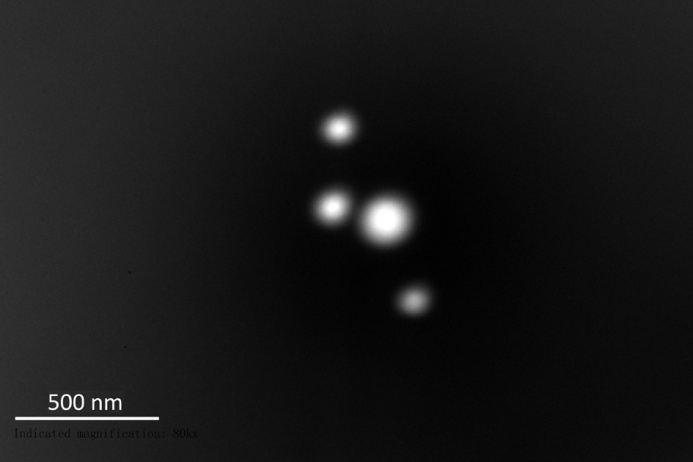


Additional File 1. TEM image of PINPs. The morphology of the PINPs was obtained by TEM (Tecnai G2 F20 U-TWIN, FEI, Hillsboro, OR, US). The scale is 500 nm.
