# Supplementary material for: Irradiation pretreatment enhances the therapeutic efficacy of platelet-membrane-camouflaged antitumor nanoparticles
Source: J Nanobiotechnology. 2020 Jul 20;18:101. doi: 10.1186/s12951-020-00660-z (PMC7372815; doi:10.1186/s12951-020-00660-z)
Supplement: Supplementary file 2 — Additional file 2. 1-N-phenylnaph-thylamine (NPN)-uptake assay. [file 12951_2020_660_MOESM2_ESM.docx]

Additional File 2

Irradiation pretreatment enhances the therapeutic efficacy of platelet-membrane-

camouflaged antitumor nanoparticles

Yin Chen^1#^, Xue Shen^2#^, Songling Han^1^, Tao Wang^1^, Jianqi Zhao^1^ , Yongwu He^1, 3^, Shilei Chen^1^, Shengqi Deng^2^, Cheng Wang^1*^ and Junping Wang^1*^

^1^ State Key Laboratory of Trauma, Burns and Combined Injury, Institute of Combined Injury

of PLA, Chongqing Engineering Research Center for Nanomedicine, College of Preventive

Medicine, Third Military Medical University, Chongqing, 400038, China

^2^ Sichuan Industrial Institute of Antibiotics, Chengdu University, Chengdu, 610106, China

^3^ College of Materials Science and Engineering, Hebei University of Engineering, Handan,

056038, China

^#^ These authors contributed equally to this work.

^*^ Corresponding authors.

Junping Wang, [wangjunping@tmmu.edu.cn](mailto:wangjunping@tmmu.edu.cn); Cheng Wang, wangctmmu@126.com.


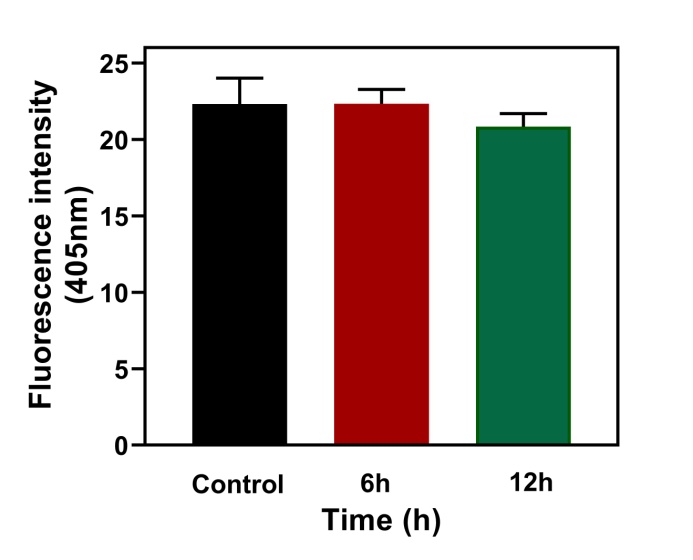


Additional File 2. 1-N-phenylnaph-thylamine (NPN)-uptake assay. NPN is a hydrophobic dye that can incorporate into impaired membranes and fluoresce upon excitation at 350 nm, exhibiting a maximum value approximately at 405 nm. 4T1 cells (1 × 10^4^ CFU) were cultured in RPMI 1640 medium containing 10% FBS in a 96-well plate for 12 h. After an irradiation of 4-Gy X-ray, the cells were further incubated for 6 and 12 h. A 10 μl aliquot of 200 μM NPN (Sigma, 104043) was added. The fluorescence intensity was measured at 405 nm with an Infinite M1000 Promicroplate reader (Männedorf, Zürich, CHE). This assay was conducted in duplicate and repeated three times.
