# Supplementary material for: Irradiation pretreatment enhances the therapeutic efficacy of platelet-membrane-camouflaged antitumor nanoparticles
Source: J Nanobiotechnology. 2020 Jul 20;18:101. doi: 10.1186/s12951-020-00660-z (PMC7372815; doi:10.1186/s12951-020-00660-z)
Supplement: Supplementary file 4 — Additional file 4. Thermal images of NaCl-treated tumor-bearing mice. [file 12951_2020_660_MOESM4_ESM.docx]

Additional File 4

Irradiation pretreatment enhances the therapeutic efficacy of platelet-membrane-

camouflaged antitumor nanoparticles

Yin Chen^1#^, Xue Shen^2#^, Songling Han^1^, Tao Wang^1^, Jianqi Zhao^1^ , Yongwu He^1, 3^, Shilei Chen^1^, Shengqi Deng^2^, Cheng Wang^1*^ and Junping Wang^1*^

^1^ State Key Laboratory of Trauma, Burns and Combined Injury, Institute of Combined Injury

of PLA, Chongqing Engineering Research Center for Nanomedicine, College of Preventive

Medicine, Third Military Medical University, Chongqing, 400038, China

^2^ Sichuan Industrial Institute of Antibiotics, Chengdu University, Chengdu, 610106, China

^3^ College of Materials Science and Engineering, Hebei University of Engineering, Handan,

056038, China

^#^ These authors contributed equally to this work.

^*^ Corresponding authors.

Junping Wang, [wangjunping@tmmu.edu.cn](mailto:wangjunping@tmmu.edu.cn); Cheng Wang, wangctmmu@126.com.


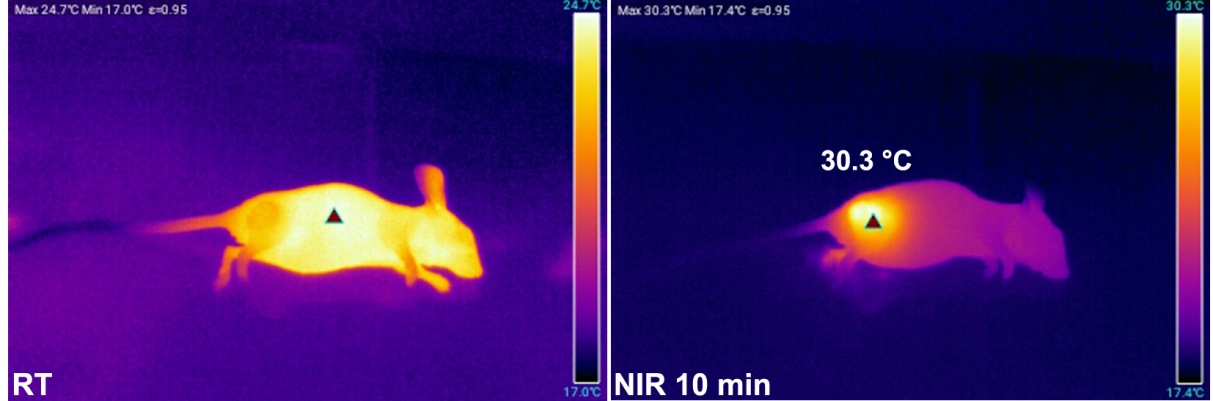


Additional File 4. Thermal images of NaCl-treated tumor-bearing mice. Female 8-week-old BALB/c mice were subcutaneously injected with 2 × 10^9^ CFU 4T1 cells in the right hind leg. When the tumor reached approximately 75 mm^3^, a total of 200 μl of NaCl solution was intravenously administered. A Fortric 226s thermal imager (Shanghai, CHN) was used to obtain the infrared thermal images and record the temperatures in the absence and presence of NIR. RT, room temperature.
