# Supplementary material for: Irradiation pretreatment enhances the therapeutic efficacy of platelet-membrane-camouflaged antitumor nanoparticles
Source: J Nanobiotechnology. 2020 Jul 20;18:101. doi: 10.1186/s12951-020-00660-z (PMC7372815; doi:10.1186/s12951-020-00660-z)
Supplement: Supplementary file 5 — Additional file 5. PM camouflage improves the antitumor ability of PINPs in vivo. [file 12951_2020_660_MOESM5_ESM.docx]

Additional File 5

Irradiation pretreatment enhances the therapeutic efficacy of platelet-membrane-

camouflaged antitumor nanoparticles

Yin Chen^1#^, Xue Shen^2#^, Songling Han^1^, Tao Wang^1^, Jianqi Zhao^1^ , Yongwu He^1, 3^, Shilei Chen^1^, Shengqi Deng^2^, Cheng Wang^1*^ and Junping Wang^1*^

^1^ State Key Laboratory of Trauma, Burns and Combined Injury, Institute of Combined Injury

of PLA, Chongqing Engineering Research Center for Nanomedicine, College of Preventive

Medicine, Third Military Medical University, Chongqing, 400038, China

^2^ Sichuan Industrial Institute of Antibiotics, Chengdu University, Chengdu, 610106, China

^3^ College of Materials Science and Engineering, Hebei University of Engineering, Handan,

056038, China

^#^ These authors contributed equally to this work.

^*^ Corresponding authors.

Junping Wang, [wangjunping@tmmu.edu.cn](mailto:wangjunping@tmmu.edu.cn); Cheng Wang, wangctmmu@126.com.


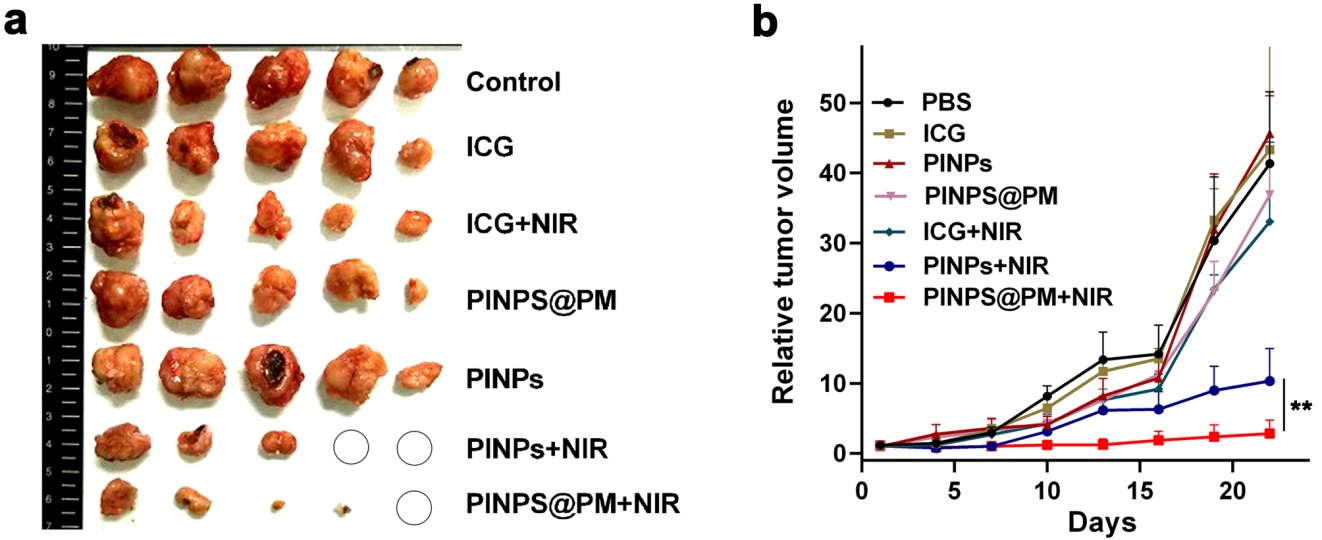


Additional File 5. PM camouflage improves the antitumor ability of PINPs *in vivo*. Female 8-week-old BALB/c mice were subcutaneously injected with 2 × 10^9^ CFU 4T1 cells in the right hind leg. PINPs@PM (60 μg, based on the content of ICG) was given by tail vein injection in the presence and absence of a 4-Gy X-ray local irradiation. NIR treatment was continued for 10 min after 24 h. The tumor volumes were monitored for three weeks. **a** Photograph of mice tumors. The circles indicate that the tumors are visually eliminated. **b** Dynamic alterations of tumor volumes. **, *P* < 0.01.
